# Trends in Antidiabetic Drug Use and Safety of Metformin in Diabetic Patients with Varying Degrees of Chronic Kidney Disease from 2010 to 2021 in Korea: Retrospective Cohort Study Using the Common Data Model

**DOI:** 10.3390/ph17101369

**Published:** 2024-10-14

**Authors:** Sung Hwan Joo, Seungwon Yang, Suhyun Lee, Seok Jun Park, Taemin Park, Sang Youl Rhee, Jae Myung Cha, Sandy Jeong Rhie, Hyeon Seok Hwang, Yang Gyun Kim, Eun Kyoung Chung

**Affiliations:** 1 Department of Regulatory Science, College of Pharmacy, Graduate School, Kyung Hee University, Seoul 02447, Republic of Korea; shsonic95@khu.ac.kr (S.H.J.); syang345@khu.ac.kr (S.Y.); psjqkr0824@khu.ac.kr (S.J.P.); ptyoon1@gmail.com (T.P.); 2 Institute of Regulatory Innovation through Science (IRIS), Kyung Hee University, Seoul 02447, Republic of Korea; 3 Department of Pharmacy, College of Pharmacy, Kyung Hee University, Seoul 02447, Republic of Korea; sh198410@khu.ac.kr; 4 Center for Digital Health, Medical Science Research Institute, College of Medicine, Kyung Hee University, Seoul 02447, Republic of Korea; rheesy@khu.ac.kr; 5 Department of Endocrinology and Metabolism, School of Medicine, Kyung Hee University, Seoul 02447, Republic of Korea; 6 Division of Gastroenterology, Department of Internal Medicine, Kyung Hee University Hospital at Gangdong, School of Medicine, Kyung Hee University, Seoul 05278, Republic of Korea; drcha@khu.ac.kr; 7 College of Pharmacy, Ewha Womans University, Seoul 03760, Republic of Korea; sandy.rhie@ewha.ac.kr; 8 Graduate School of Pharmaceutical Sciences, Ewha Womans University, Seoul 03760, Republic of Korea; 9 Division of Nephrology, Department of Internal Medicine, Kyung Hee University Hospital, Kyung Hee University College of Medicine, Seoul 02447, Republic of Korea; 10 Division of Nephrology, Department of Internal Medicine, College of Medicine, Kyung Hee University, Seoul 02447, Republic of Korea; 11 Department of Pharmacy, Kyung Hee University Hospital at Gangdong, Seoul 05278, Republic of Korea

**Keywords:** chronic kid-ney disease, type 2 diabetes mellitus, observational medical outcomes partnership (OMOP), common data model (CDM), real-world data (RWD), metformin-associated lactic acidosis, pharmacoepidemiology, pharmacovigilance

## Abstract

Background/Objectives: This study aimed to investigate trends in antidiabetic drug use and assess the risk of metformin-associated lactic acidosis (MALA) in patients with chronic kidney disease (CKD). Methods: A retrospective observational analysis based on the common data model was conducted using electronic medical records from 2010 to 2021. The patients included were aged ≥18, diagnosed with CKD and type 2 diabetes, and had received antidiabetic medications for ≥30 days. MALA was defined as pH ≤ 7.35 and arterial lactate ≥4 mmol/L. Results: A total of 8318 patients were included, with 6185 in CKD stages 1–2 and 2133 in stages 3a–5. Metformin monotherapy was the most prescribed regimen, except in stage 5 CKD. As CKD progressed, metformin use significantly declined; insulin and meglitinides were most frequently prescribed in end-stage renal disease. Over the study period, the use of SGLT2 inhibitors (13.3%) and DPP-4 inhibitors (24.5%) increased significantly, while sulfonylurea use decreased (*p* < 0.05). Metformin use remained stable in earlier CKD stages but significantly decreased in stage 3b or worse. The incidence rate (IR) of MALA was 1.22 per 1000 patient-years, with a significantly increased IR in stage 4 or worse CKD (*p* < 0.001). Conclusions: Metformin was the most prescribed antidiabetic drug in CKD patients in Korea with a low risk of MALA. Antidiabetic drug use patterns varied across CKD stages, with a notable decline in metformin use in advanced CKD and a rise in SGLT2 inhibitor prescriptions, underscoring the need for further optimized therapy.

## 1. Introduction

Chronic kidney disease (CKD) is a common complication in patients with diabetes mellitus (DM) [1,2]. The prevalence of CKD in diabetic patients was >25% in 2014, and 40% of patients with type 2 DM are likely to have CKD during their lifetime [3]. Kidney diseases in patients with DM are associated with substantial morbidity and mortality, including an increased risk for atherosclerotic cardiovascular disease and, ultimately, premature mortality [1]. Globally, an estimated 2 million deaths occurred due to DM and CKD [4] in 2019. In Korea, DM was the most common cause of end-stage renal disease (ESRD) in 2021, accounting for 47% of all ESRD cases [5]. Additionally, the pharmacologic management of type 2 DM is further complicated by CKD due to a substantial decline in kidney function, leading to impaired clearance of antidiabetic medications [6]. Consequently, diabetic patients with CKD are more susceptible to adverse drug reactions, such as hypoglycemia and metformin-associated lactic acidosis (MALA) [7,8].

Historically, metformin (MET) has been suggested as the first-line medication for the management of type 2 DM due to its glucose-lowering efficacy and tolerable safety profile [9]. In patients with CKD, its use had not been recommended due to the potential risk of MALA [10]. However, the risk of MALA has been considered low, with its incidence ranging from 3 to 10 per 100,000 person-years in patients with an estimated glomerular filtration rate (eGFR) of 30 to 60 mL/min/1.73 m^2^ [10]. More recently published studies have suggested a significantly increased risk of MALA in patients with eGFR < 30 mL/min/1.73 m^2^ [11,12,13]. Therefore, according to current practice guidelines for the management of type 2 DM, as well as dosing information approved by regulatory agencies such as the Food and Drug Administration (FDA), MET is contraindicated in patients with eGFR < 30 mL/min/1.73 m^2^, with its initiation not recommended in those with eGFR between 30 and 44 mL/min/1.73 m^2^ [6]. Recently, sodium-glucose cotransporter-2 inhibitors (SGLT2is) and certain glucagon-like peptide 1 receptor agonists (GLP-1RAs) were recommended as first-line treatments, along with MET, in CKD patients due to their nephroprotective and cardiovascular benefits.

Despite the established guidelines [6,9], substantial variability in the prescription of antidiabetic drugs for CKD patients has been reported in clinical practice, potentially due to demographic, clinical, and sociocultural factors of drug therapy [14]. Indeed, MET has continued to be prescribed to a substantial proportion of patients with eGFR < 30 mL/min/1.73 m^2^ [11,12,13], including ESRD [15,16,17], while less than one-third of diabetic patients with CKD initially received SGLT2is therapy [18]. Therefore, the trends and safety of pharmacotherapy for diabetic patients with CKD in real-world clinical practice should be evaluated using real-world data (RWD). Among RWD, electronic medical records (EMRs) are the representative type of RWD containing laboratory data and thus are frequently used for pharmacoepidemiology, as well as pharmacovigilance research requiring laboratory data to define patient cohort and outcomes (e.g., CKD, lactic acidosis). However, EMR-based research requires substantial time and effort for data collection and analysis, especially when multiple institutions are involved or EMRs over a long period of time are reviewed [19]. To address these problems associated with EMR-based clinical research, common data models (CDMs) have emerged as a common and simple EMR model to facilitate clinical studies [20]. A CDM transforms EMR data from disparate sources into a common format with consistent definitions and structures, thereby enabling more efficient data integration, query, and analysis. Although the EMR data transformation may not be comprehensive, this standardization facilitates multi-institutional research and supports a wide range of research applications. Importantly, it emphasizes the anonymization of patient data, making it impossible to track individual patients, thereby enhancing the privacy and security of the data used in studies [21,22].

The patterns of medication use, as well as the risk of adverse drug reactions, might vary in different countries or ethnic groups due to demographic, clinical, and sociocultural differences; however, there is a relative paucity of data regarding the patterns of antidiabetic medication use, as well as the risk of MALA, in patients with varying stages of CKD in the real world, particularly in Korea, through the assessment of objective laboratory data. Therefore, the aims of this study were to examine trends of antidiabetic drug use and evaluate the risk of MALA in patients with CKD in real-world clinical practice by analyzing the EMR-based CDM data over 12 years.

## 2. Results

### 2.1. Patient Characteristics

**Figure 1:**
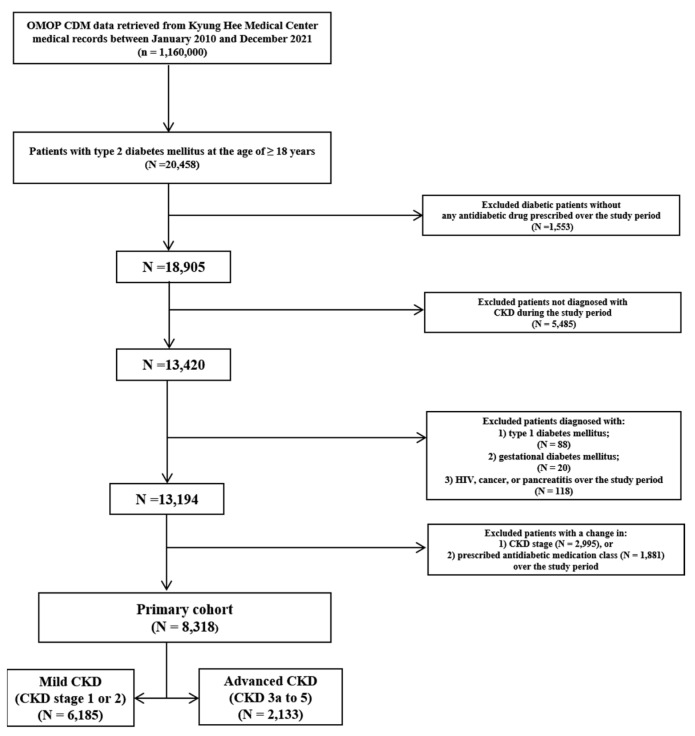
Flowchart of study patient selection. OMOP CDM, the observational medical outcomes partnership common data model; CKD, chronic kidney disease.

Among a total of 20,458 adult patients diagnosed with type 2 DM over the study period, 13,420 patients diagnosed with CKD, as well as type 2 DM, receiving ≥1 antidiabetic drug were identified; 2995 (22.3%) and 1881 (14.0%) patients were excluded due to a change in CKD stage and prescribed antidiabetic drug class, respectively. After applying the inclusion and exclusion criteria (Figure 1), overall, 8318 patients (n = 6185 in mild CKD; n = 2133 in advanced CKD) were eligible for this study.

The baseline characteristics of our study patients are summarized in Table 1. Compared to patients with mild CKD, those with advanced CKD were older (60.6 ± 12.2 to 69.8 ± 8.9 vs. 58.9 ± 11.1 years); received more concurrent medications, especially those for the treatment of cardiovascular diseases, including angiotensin-converting enzyme inhibitors (ACEIs), angiotensin receptor blockers (ARBs), beta-blockers, diuretics, calcium channel blockers, and antiplatelets; and had a higher prevalence of comorbid cardiovascular diseases, particularly hypertension, with a higher Charlson comorbidity index (CCI) score (2.4 ± 1.5 to 3.7 ± 1.5 vs. 2.2 ± 1.4).

### 2.2. Patterns of Antidiabetic Medication Use in Chronic Kidney Disease

**Figure 2:**
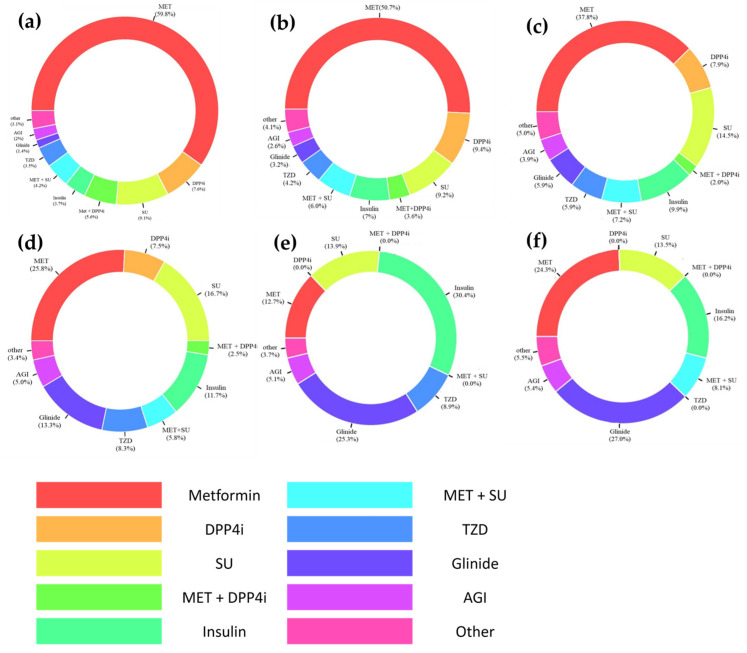
Patterns of prescribing antidiabetic drug regimen in patients with chronic kidney disease (CKD) by stages: (**a**) mild CKD (i.e., stage 1 to 2); (**b**) CKD stage 3a; (**c**) CKD stage 3b; (**d**) CKD stage 4; (**e**) CKD stage 5 receiving dialysis; and (**f**) CKD stage 5 not receiving dialysis. Abbreviations: AGI, alpha-glucosidase inhibitor; DPP4i, dipeptidyl peptidase-4 inhibitor; Glinide, meglitinide; MET, metformin; SU, sulfonylurea; TZD, thiazolidinedione.

The patterns of using antidiabetic treatment regimens in patients with CKD are described in Figure 2. The most frequently prescribed antidiabetic medication regimen was MET monotherapy in patients with mild CKD (stage 1 to 2; 59.8%), as well as in those with CKD stage 3a (50.7%). The prevalence of MET monotherapy significantly decreased as CKD progressed to stage 3b or higher (*p* < 0.001); however, MET was still substantially used even in patients with ESRD (12.7% with dialysis therapy; 32.4% without dialysis therapy), as well as in those with CKD stage 4 (34.1% including both monotherapy and combination therapy). As CKD progressed, insulin and meglitinides (glinides) were more frequently prescribed (*p* < 0.001). In patients with ESRD, insulin monotherapy was most prevalent among those on dialysis, followed by glinide monotherapy; for those not on dialysis, glinide monotherapy was most frequently prescribed, followed by MET and insulin monotherapy. Across CKD stages, SGLT2is or GLP-1RAs were used in very few patients over the study period.

**Figure 3:**
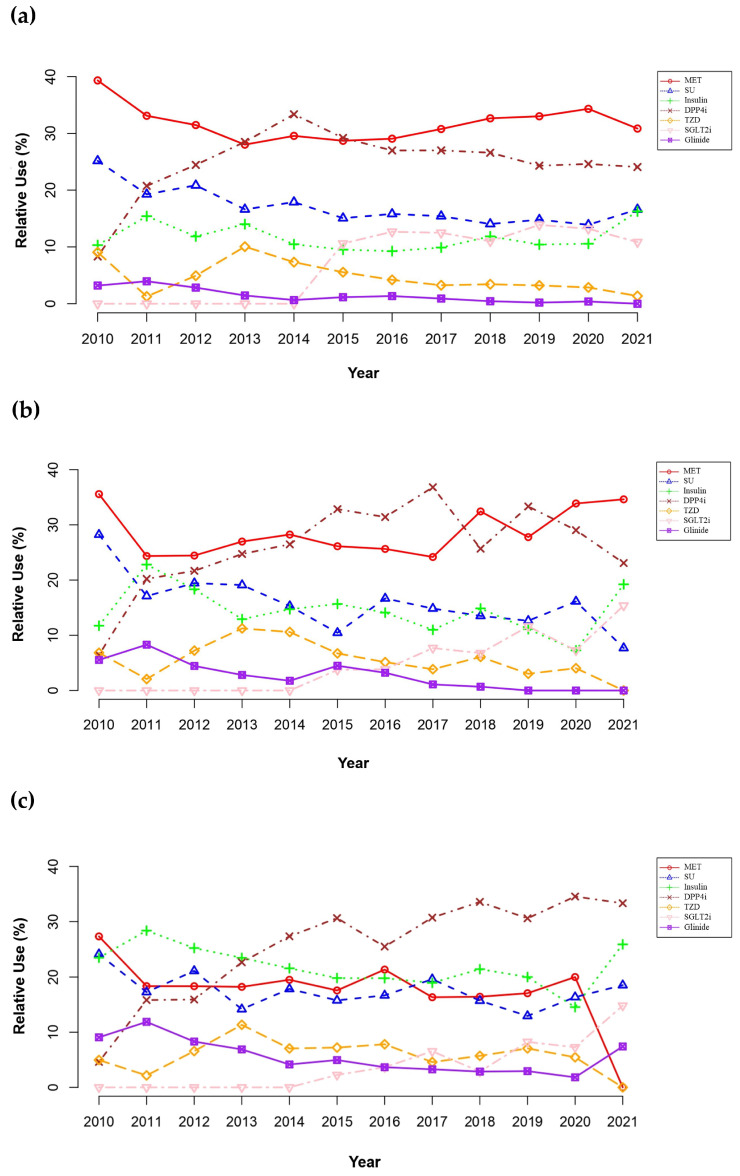
Temporal trends of prescribing antidiabetic drugs in patients with chronic kidney disease (CKD) by stages: (**a**) mild CKD (i.e., stage 1 to 2); (**b**) CKD stage 3a; (**c**) CKD stage 3b to 5. Alpha-glucosidase inhibitor is not shown on the plot due to minimal use throughout the study period. Abbreviations: DPP4i, dipeptidyl peptidase-4 inhibitor; glinide, meglitinide; MET, metformin; SGLT2i, sodium-glucose cotransporter-2 inhibitor; SU, sulfonylurea; TZD, thiazolidinedione.

Over the study period, the prescribing patterns of antidiabetic drugs were significantly changed in all CKD stages (Figure 3; Supplementary Table S1). The use of sulfonylureas (SUs) as a proportion of overall antidiabetic medication use declined across CKD stages (decrease by 12.4%; *p* < 0.05), while no patients received GLP-1RAs. Notably, despite stable or moderately decreased use of other antidiabetic drug classes over the study period, the use of SGLT2is and dipeptidyl peptidase-4 inhibitors (DPP4is) increased significantly (increase by 13.3% in SGLT2i use; 24.5% in DPP4i use; *p* < 0.05). During the study period, the overall use of MET remained stable in patients with mild CKD (28.4% to 40.6%, *p* > 0.05) or those with CKD stage 3a (25.6% to 37.6%; *p* > 0.05). However, its use was significantly reduced in patients with CKD stage 3b or worse (30.1% in 2010 to 0.0% in 2021; *p* < 0.05).

In patients with mild CKD (Figure 3a), MET was the dominant antidiabetic in 2010 but decreased from 40.6% in 2010 to 28.4% in 2013 with an increase in DPP4i use (8.6% in 2010 to 28.9% in 2013); the use of DPP4is surpassed MET in 2013 and 2014. Afterward, the use of DPP4is declined from a peak of 33.6% in 2014 to 24.1% in 2021 with a substantial increase in SGLT2i use during the same period (0.0% in 2014 to 10.8% in 2021).

Similarly, patients with CKD stage 3a (Figure 3b) were most frequently prescribed MET in 2010. However, its use substantially decreased from 37.6% in 2010 to 24.4% in 2017, with an increase in DPP4i use (6.8% in 2010 to the peak of 37.2% in 2017); the use of DPP4is surpassed MET from 2015. Afterward, a decrease in prescribing DPP4is was observed (37.2% in 2017 to 23.1% in 2021), with a gradual increase in using MET (24.4% in 2017 to 34.6% in 2021) and SGLT2is (7.8% in 2017 to 15.4% in 2021).

For advanced CKD stage 3b or worse (Figure 3c), MET was the most commonly prescribed antidiabetic drug in 2010. However, its use was substantially reduced from 30.1% in 2010 to none in 2021, with a substantial increase in the use of DPP4is (5.1% in 2010 to 36.0% in 2021); the use of DPP4is surpassed MET from 2013. A remarkable reduction in MET use was observed in 2021 (20.4% in 2020 to 0.0% in 2021), with substantial increases in prescribing SGLT2is (7.4% in 2020 to 16.0% in 2021), as well as insulin (14.8% in 2020 to 29.0% in 2021) and glinides (1.8% in 2020 to 7.4% in 2021).

### 2.3. Risk of Metformin-Associated Lactic Acidosis in Chronic Kidney Disease

The incidence rates of MALA before and after propensity score matching (PSM) are summarized in Table 2. No patient experienced multiple events of MALA over the study period. Overall, the crude incidence rate of MALA was 1.22 (95% confidence interval [CI], 0.80–1.77) per 1000 patient-years, with 27 cases occurring over the study period; the incidence rate of MALA was higher in patients with advanced CKD compared to those with mild CKD. After PSM, the adjusted incidence rate of MALA was 1.35 (95% CI, 0.16–4.90) per 1000 patient-years, with only two cases identified; the adjusted incidence rate of MALA was not estimated in patients with CKD stage 3b or worse due to a small number of patients left after PSM.

Fatal cases of MALA occurred in nine patients (0.1%), as follows: three (0.4%) in CKD stage 3a, three (0.7%) in CKD stage 3b, one (0.7%) in CKD stage 4, and two (1.9%) in CKD stage 5. No fatalities occurred in patients with mild CKD. Overall, the crude incidence rate of fatal MALA was 0.40 (95% CI, 0.18–0.76) per 1000 patient-years, with a higher incidence rate at advanced CKD stages (1.26 [95% CI, 0.26–3.70] in CKD stage 3a; 2.38 [0.49–6.97] in CKD stage 3b; 2.57 [0.06–14.3] in CKD stage 4; and 7.93 [0.96–28.7] in CKD stage 5). 

## 3. Discussion

To our knowledge, this is one of the first few studies utilizing the observational medical outcomes partnership common data model (OMOP CDM) based on EMRs to evaluate the prevalence and safety of antidiabetic medication use in patients with CKD. The OMOP CDM was an appropriate RWD source for our current study due to its comprehensive inclusion of EMR elements including laboratory test results (e.g., kidney function, serum lactate concentrations), enhancing the efficiency of extracting large-volume EMR data [23]. Similar to previous studies using various sources of RWD [24,25,26,27], our study suggested that the majority of patients have comorbid cardiovascular diseases, such as hypertension, and receive cardiovascular medications, including ACEIs or ARBs (Table 1). Overall, MET monotherapy was most frequently prescribed to patients with CKD; however, its use substantially declined at advanced CKD stages, with the insulin- and glinide-based therapy increasing (Figure 2). The incidence of MALA was relatively low, with an even lower incidence of fatal MALA (Table 2). With the availability of newer antidiabetic medications, as well as novel clinical trial findings [28,29,30,31], the prescribing trends of antidiabetic drugs to patients with CKD changed gradually over the study period (Figure 3).

Consistent with previous studies [24,25,26,32,33,34,35], our study suggested MET monotherapy as the most frequently prescribed antidiabetic regimen for CKD patients, decreasing significantly as the CKD stage advanced in favor of insulin and glinides (Figure 2). Similar prevalence rates of MET usage in early CKD stages (1 to 3a) were noted compared to previous studies (50.7–59.8% vs. 35.5–67.3% in previous studies) [25,26,34]. However, our present study suggested a substantially higher proportion of MET use in patients with CKD stage 4 or higher (12.7–25.8% vs. <5%) [25,26,34]. The higher prevalence of MET use might be associated with the national health insurance reimbursement policy in Korea during the study period; MET- or SU-based regimens should be prescribed prior to adding or switching to other antidiabetic medications [36]. In addition, considering the perceived low risk of MALA [37], patients with advanced CKD (i.e., stage 4 or above) might continue to receive MET if their glycemic control was stabilized, particularly those initiated with MET before coming to KHMC. In contrast to MET, SGLT2is and DPP4is were used much less in our study compared to previous studies (SGLT2is: <5% vs. 9.4–17%; DPP4is: 7.5–9.4% vs. 10.5–28.3%) [25,26,34], the cause of which was potentially influenced by the aforementioned national health insurance reimbursement policy for antidiabetic medications [36]. In patients with ESRD, insulin and glinides were used most commonly in our current study (Figure 2) because many antidiabetic drugs are considered inappropriate due to lower efficacy (e.g., SGLT2is) and/or a higher risk of adverse reactions (e.g., MALA), leading to a marked increase in using insulin and glinides in our patients with ESRD.

Over the study period, cardinal events related to antidiabetic therapy occurred, resulting in substantial changes in prescribing trends of antidiabetic medications (Figure 3). Novel antidiabetic drug classes, including SGLT2is and GLP-1RAs, were first approved in 2013 and 2015, respectively, in Korea [28]. In addition, new findings from breakthrough clinical trials, such as the EMPA-REG OUTCOME, CREDENCE, and DAPA-CKD trials, were published regarding the renal and cardiovascular outcomes of antidiabetic therapy in patients with CKD [29,30,31]. Overall, our current study showed similar temporal trends of antidiabetic drug use compared to previous studies [25,26,38]. Due to the risk of MALA, as well as the availability of novel and safe antidiabetic drugs (e.g., DPP4is, SGLT2is) in patients with CKD, MET use substantially declined during the initial study period. However, since 2017, the prevalence of prescribing MET has gradually increased and returned near the baseline, potentially because of the changes in the approved label of MET. The approved MET label was revised based on the best available scientific evidence at the end of 2016 to allow MET use in patients with eGFR > 30 mL/min/1.73 m^2^ [17]. In our present study, none of the patients with eGFR < 45 mL/min/1.73 m^2^ received MET in 2021, potentially because of replacing its use with SGLT2is, as well as insulin and glinides. The remarkable increase in prescribing SGLT2is in 2021 might be associated with the updates in the guideline recommendations of the ADA and the Korean Diabetes Association, suggesting SGLT2is as the first-line agent in diabetic patients with CKD due to their renal benefits [39,40]. Nephroprotective effects of SGLT2is were reported with empagliflozin, canagliflozin, and dapagliflozin in 2016, 2019, and 2020, respectively, contributing to updated guideline recommendations [29,30,31]; thus, the use of SGLT2is substantially increased. Consistent with previous studies [25,26,38], the use of DPP4is substantially increased over the study period, particularly in patients with eGFR < 45 mL/min/1.73 m^2^, as they were considered safe and effective therapeutic alternatives in patients with CKD. However, despite the established cardiovascular and renal benefits of GLP-1RAs [6,41], none of our study patients were prescribed GLP-1RAs, which is possibly due to the national health insurance reimbursement policy [36]; in Korea, GLP-1RAs have been covered by national health insurance only in combination with either concurrent use of MET and SU or insulin. Consequently, their therapeutic places might be inappropriately replaced by other hypoglycemic agents such as glinides. Therefore, our analysis of temporal trends in prescribing antidiabetic medications in patients with CKD demonstrated the importance of timely updates of national and/or institutional policy on medication use to implement the best available evidence in practice.

MET is more closely associated with MALA than other antidiabetic medications due to its pharmacokinetic and metabolic effects. MET inhibits hepatic gluconeogenesis and increases anaerobic glucose metabolism, leading to lactate accumulation, particularly when renal function is impaired and clearance is reduced. In patients with CKD, reduced renal function leads to accumulation of MET, increasing the risk of lactic acid production [42,43]. Despite the substantial use of MET, the overall risk of MALA was considered relatively low in our present study (Table 2). Similar to our study, previous studies reported a low risk of lactic acidosis with MET [10,37,44]; the overall prevalence of MALA was suggested to be much lower (>20-fold difference) than that of lactic acidosis associated with phenformin [45], which was withdrawn from the market in the late 1970s owing to the risk of lactic acidosis [46]. Previous studies on the benefits and harms of antidiabetic drugs have confirmed the low incidence of renal-related adverse outcomes, such as MALA [47], underscoring its safety when used according to renal function-adjusted guidelines [9]. However, other antidiabetic medications, such as SGLT2is and GLP-1RAs, were considered safer and more effective compared to MET, implying more preferred treatment options to MET [47]. This aligns with our findings that suggest a cautious application of MET in CKD stages 3 to 5 due to its potential risks of lactic acidosis, emphasizing the need for alternative treatments in patients with advanced kidney impairment. Our estimated incidence of MALA was within the previously reported range, but in the upper end (<1 to 138/100,000 patient-years) [48,49]. The relatively high incidence of MALA in our current study might be accounted for by substantial renal impairment in all of our study patients, with ≥25% at advanced CKD stages, treated with MET at relatively high doses (i.e., ≥1000 mg daily) chronically (i.e., ≥30 days) [37,44,50]. In fact, a recent retrospective single-center study conducted at a tertiary acute-care teaching hospital showed a similar risk of MALA as our study (19 patients with seven fatal cases over 9 years) in patients with substantial impairment of kidney function, including ESRD [51]. Consistent with previous studies [43,49,52], the risk of lactic acidosis increased at advanced CKD stages (Table 2), which confirms the approved prescribing information of MET, as well as guideline recommendations to avoid MET in patients with eGFR < 30 mL/min/1.73 m^2^, preferably no MET initiation in patients with eGFR < 45 mL/min/1.73 m^2^ [6,28,53]. Nonetheless, MET has been substantially prescribed to patients with CKD stage 3b or worse, potentially resulting in an increased risk of MALA including fatal cases [8,25]. Interventions to improve the appropriateness of MET use might need to be implemented in clinical practice, including clinical decision support systems. Overall, MET could be safely used in patients with mild to moderate CKD with a low risk of MALA; however, extreme caution needs to be exercised in patients with moderate to severe CKD due to a substantially increased risk of MALA.

There are study limitations to be addressed. First, due to the retrospective observational nature of our current study, PSM was conducted between MET-treated and non-MET-treated patient groups to estimate the adjusted incidence rate of MALA. A substantial imbalance in sample size between the two groups was noted because MET was the most prevalent antidiabetic medication used as either monotherapy or combination therapy; consequently, the post-PSM sample size was insufficient to estimate the adjusted incidence rate with robustness, particularly in patients with CKD stage 3b or worse, to whom MET was much less frequently prescribed. Furthermore, the rarity of MALA poses an additional challenge to the robustness of PSM, which was the only adjustment method available for the estimation of incidence rates in the ATLAS analytical tool for our OMOP CDM database. Future studies may be warranted, with multi-institutional CDM containing a sufficient number of MALA cases, to better estimate adjusted incidence rates and risk with more robust adjustment methods. Additionally, as EMR-based CDM data were utilized in our present study, we could not include any events or encounters not documented in the EMR-based CDM database of our institution. In this context, the national health insurance claims data might be useful; however, the claims data do not contain relevant clinical measurements, such as laboratory test results. Thus, the claims data could only identify lactic acidosis cases through documented diagnostic codes or procedure-based operational definitions without considering objective laboratory data, which could result in an underestimation of MALA incidence. As our current study defined MALA based on objective measurements using arterial blood gas test results in the EMR-based CDM database, the risk of MALA might be more accurately captured. Owing to the difficulty in accessing multi-institution EMR-based CDM databases, our current study was performed by analyzing the single-center CDM database at our institution (i.e., KHMC). To ensure sufficient sample size, variables with substantial missing data were not included as a cohort or outcome definition such as urinary albumin excretion for CKD definition; although albuminuria should be evaluated for accurate staging of CKD, the urinary albumin excretion or albuminuria diagnostic code was substantially missing in our CDM database. Since our data extended back to 2010, earlier CKD guidelines, such as the 2012 KDIGO guidelines, were applicable, in which albuminuria was not significantly considered to define CKD stages [2]. Nevertheless, to further enhance study robustness, it is crucial to evaluate the impact of longitudinal, progressive changes in GFR or albuminuria on treatment patterns and outcomes in patients with CKD. Lastly, the use of CDM based on the ATLAS tool did not allow us to follow up on individual patient cases, i.e., changes in treatment or procedures as the CKD stage progresses, changes in therapy after the occurrence of MALA, etc.

## 4. Methods

### 4.1. Data Source

This retrospective observational study was performed by using a clinical database of Kyung Hee Medical Center (KHMC; Seoul, Republic of Korea) that was extracted, transformed, and loaded by the OMOP CDM, version 5.3 [54]. Kyung Hee Medical Center is a tertiary acute-care hospital affiliated with Kyung Hee University. In this study, the OMOP CDM of KHMC over the period of January 2010 to December 2021 was reviewed, containing >73 million medical records from 1.16 million patients. This study was approved by the institutional review board at KHMC (IRB No. KHIRB-MED-MDB-07-017), and written informed consent was exempted by the board.

### 4.2. Study Population

Included patients were those aged 18 years or older who were diagnosed with CKD, as well as type 2 DM, and received at least one antidiabetic drug. In our patient cohort, the index start date was defined as the first date of antidiabetic drug prescription with ≥ 365 days of previous clinical observation history documented in the database. The cohort exit date was the date of last documented observation in the database before study termination (i.e., 31 December 2021). Excluded patients were those with a change in prescribed antidiabetic drug class or CKD stage or those diagnosed with gestational or type 1 DM, cancer, human immunodeficiency virus (HIV) infection, or pancreatitis over the study period [55,56]. Patients with a change in prescribed antidiabetic drug class or CKD stage were excluded due to the inherent limitations of OMOP CDM and its analytical tool, ATLAS [21]; individual patient records could not be followed over time (e.g., change in medication use after a change in CKD stage or the occurrence of MALA), but only the population statistics of variables of interest were shown.

Eligible patients were classified into the following two groups: mild vs. advanced CKD groups. The mild CKD group was defined as patients with the diagnostic code for CKD stage 1 or 2 or those with a CKD diagnosis and ≥2 consecutive eGFRs of ≥60 mL/min/1.73 m^2^ measured at >3-month intervals [2,24,32,33]. Advanced CKD group was defined as patients with the diagnostic codes for CKD stage 3, 4, or 5; those with a CKD diagnosis and ≥2 consecutive eGFRs of <60 mL/min/1.73 m^2^ measured at >3-month intervals; or those receiving dialysis therapy [2,24,32,33]. The glomerular filtration rate was estimated using the Modification of Diet in Renal Disease (MDRD) study equation [57,58]. Baseline characteristics were extracted at the index start date, including age, sex, CCI score, comorbid diseases based on documented diagnostic codes, and concomitant medications.

### 4.3. Trends in Antidiabetic Medication Use

Antidiabetic medications were classified into the following groups based on their pharmacologic classes: biguanide (i.e., MET), SU, DPP4i, thiazolidinedione (TZD), alpha-glucosidase inhibitor (AGI), glinide, insulin, SGLT2i, and GLP-1RA. The antidiabetic drug regimen was categorized into either monotherapy or combination therapy. Monotherapy was defined as using a single antidiabetic medication in a specific antidiabetic drug class. Combination therapy was defined as using ≥2 drugs in different classes. Change in antidiabetic drug use for exclusion was defined as (1) adding ≥1 antidiabetic medication in a class different from drugs contained in the prescribed antidiabetic drug regimen, (2) removing ≥1 antidiabetic medication from the prescribed treatment regimen, or (3) replacing ≥1 antidiabetic medication in the prescribed treatment regimen with a drug(s) in a different class.

The patterns of using antidiabetic therapy were characterized by antidiabetic treatment regimens based on medication classes using sunburst plots through the ATLAS interactive analysis platform (version 2.7.6, Observational Health Data Sciences and Informatics [OHDSI]). While utilizing the ATLAS platform enhances accessibility to data analysis, this tool restricts the customization of analysis methods [21]. Antidiabetic medications prescribed for continuous use of ≥30 days were included in the analysis. Antidiabetic drug regimens used in <5 cases were excluded.

### 4.4. Safety of Metformin Use

The safety of MET use in diabetic patients with CKD was assessed based on the risk of MALA. Lactic acidosis was defined as pH ≤ 7.35 and blood arterial lactate≥ 4 mmol/L based on arterial blood gas test results [59]. MALA was defined as the occurrence of lactic acidosis following ≥1 dose of MET administered to study patients. Among MALA events, fatal cases were defined as those resulting in death during hospitalization after the onset of MALA [60].

### 4.5. Statistical Analysis

All statistical analyses were performed using R (version 4.2.1, R Core Team 2022, Vienna, Austria), as well as ATLAS (version 2.7.6, OHDSI), which is the web-based interactive analysis platform for OMOP CDM. Descriptive statistics were used to summarize continuous variables in mean ± standard deviation (SD) and categorical variables in counts with proportions (%). Patient characteristics were compared statistically among mild CKD and CKD stage 3a, 3b, 4, and 5 groups. For continuous variables, normality and homoscedasticity assumptions were tested using the Kolmogorov–Smirnov test and Bartlett’s test, respectively. Because the normality assumption was violated for all continuous variables, the Kruskal–Wallis test with a post hoc Dunn–Bonferroni analysis was performed to test the differences among patient groups. For categorical variables, a χ^2^ or Fisher exact test was used for statistical comparison among patient groups.

The trends of antidiabetic medication use over time were comparatively assessed for each pharmacological drug class using the Cochran–Armitage trend test in mild CKD, CKD stage 3a, and CKD stage 3b or worse based on recommended dosing adjustment groups in approved prescribing information [28,32,33,53]. The crude incidence of MALA per 1000 patient-years was estimated as follows (Equation (1)):

(1) $$\frac{Total\;number\;of\;MALA\;event\;occurrences\;during\;the\;study\;period}{Person-time\;over\;the\;study\;period\;\left(years\right)}\times 1000$$

An exploratory analysis was performed to compare the crude incidence rate ratios across CKD stages using Fisher’s exact tests, with mild CKD (i.e., CKD stage 1 to 2) as a reference group. To control for different patient characteristics, PSM was performed between MET- and non-MET-treated patient groups. Patients were matched at a ratio of 1:1 using propensity scores based on multivariable logistic regression for baseline characteristics, including age, sex, CCI, concurrently used medications, and comorbid diseases. The adjusted incidence rate of MALA was estimated after PSM. Subgroup analyses were performed to account for the effects of different CKD stages (i.e., CKD stage 3a, 3b, 4, and 5) on the risk of MALA. Statistical significance was defined as *p* < 0.05.

## 5. Conclusions

Our present study utilized the EMR-based CDM database over 12 years to assess trends in antidiabetic drug use and evaluate the risk of MALA in patients with CKD. Overall, patterns of antidiabetic medication use were substantially varied at different CKD stages over the study period. MET monotherapy was predominantly prescribed to patients with mild to moderate CKD stages; however, its use significantly declined at advanced stages, with a significant increase in prescribing insulin and glinides. Very few patients received SGLT2is or GLP1-RAs, which are the guideline-recommended first-line antidiabetic medications for diabetic patients with CKD. The use of SGLT2is has gradually increased since 2015; however, no patient received GLP1-RAs over the study period. The overall use of MET was stable; a substantial proportion of patients across all CKD stages received MET. Nonetheless, the overall risk of MALA was considered low. Advanced CKD stages were associated with an increase in the risk of MALA, particularly in patients with CKD stage 4 or worse. Considering the relative benefits and risks, antidiabetic medication therapy should be further optimized in patients with varying degrees of CKD. Additional caution should be exercised for the safe and effective use of antidiabetic drugs in patients with advanced CKD stages, such as CKD stage 4 or worse, through close monitoring.

## Figures and Tables

**Table 1:** Baseline characteristics of CKD patients treated with antidiabetic medications from 2010 to 2021 ^a^.

CKD Group or Stage	Mild CKD ^b^ (n = 6185)	Advanced CKD ^c^ (n = 2133)					
		CKD 3a (n = 974)	CKD 3b (n = 587)	CKD 4 (n = 252)	CKD 5		*p*-Value ^d^
					Without Dialysis (n = 96)	With Dialysis (n = 224)	
Age (years)	58.9 ± 11.1	67.8 ± 9.1	69.8 ± 8.9	69.6 ± 10.4	66.7 ± 11.2	60.6 ± 12.2	<0.001
CCI score	2.2 ± 1.4	2.4 ± 1.5	2.8 ± 1.8	3.0 ± 1.7	3.3 ± 1.7	3.7 ± 1.5	<0.001
Female	3881 (62.7)	292 (30.0)	196 (33.4)	103 (40.9)	41 (42.7)	75 (33.5)	<0.001
**Comorbidities**							
HTN	4016 (64.9)	775 (79.6)	492 (83.8)	213 (84.5)	79 (82.3)	182 (81.2)	0.005
Dyslipidemia	4701 (76.0)	657 (67.5)	363 (61.8)	144 (57.1)	47 (49.0)	107 (47.8)	<0.001
CHF	166 (2.7)	51 (5.2)	33 (5.6)	15 (6.0)	6 (6.2)	16 (7.1)	0.82
CAD	143 (2.3)	36 (3.7)	33 (5.6)	13 (5.2)	3 (3.1)	11 (4.9)	0.83
COPD	124 (2.0)	40 (4.1)	32 (5.5)	12 (4.8)	3 (3.1)	6 (2.7)	0.78
**Concurrent medications**							
ACEIs	497 (8.0)	107 (11.0)	86 (14.7)	62 (24.6)	19 (19.8)	62 (27.7)	0.003
ARBs	3609 (58.4)	726 (74.5)	463 (78.9)	206 (81.7)	82 (85.4)	193 (86.2)	<0.001
BBs	1738(28.0)	366 (37.6)	248 (42.2)	129 (51.1)	50 (52.1)	145 (64.7)	<0.001
Diuretics	1657 (26.8)	449 (46.1)	361 (61.5)	197 (78.1)	81 (84.4)	201 (89.7)	<0.001
CCBs	3299 (53.3)	673 (69.1)	451 (76.8)	209 (82.9)	87 (90.6)	216 (96.4)	<0.001
Digoxin	67 (1.1)	36 (3.7)	35 (6.0)	23 (9.1)	7 (7.3)	15 (6.7)	0.175
Aspirin	2441 (39.5)	561 (57.6)	362 (61.7)	160 (63.5)	28 (29.0)	163 (72.8)	<0.001
Antiplatelet agents other than aspirin	969 (15.7)	195 (20.0)	150 (25.6)	87 (34.5)	39 (40.6)	207 (92.4)	<0.001
Nitrates	135 (2.2)	73 (7.5)	52 (8.9)	30 (11.9)	5 (5.2)	39 (17.4)	0.035
Warfarin	72 (1.2)	40 (4.1)	37 (6.3)	24 (9.5)	7 (7.3)	13 (5.8)	0.185
Anticoagulant agents other than warfarin	162 (2.6)	75 (7.7)	50 (8.5)	25 (9.9)	3 (3.1)	11 (4.9)	0.143
Statins	5300 (85.7)	802 (82.3)	475 (80.9)	200 (79.4)	74 (77.1)	184 (82.1)	0.742

^a^ Data are presented as mean ± standard deviation or count (%). ^b^ Mild CKD is defined as CKD stage 1 to 2. ^c^ Advanced CKD is defined as CKD stage 3a to 5. ^d^ *p*-value for comparison among mild CKD, CKD 3a, CKD 3b, CKD 4, and CKD 5 without dialysis and CKD 5 with dialysis. Abbreviations: CKD, chronic kidney disease; CCI, Charlson comorbidity index; HTN, hypertension; CHF, chronic heart failure; CAD, coronary artery disease; COPD, chronic obstructive pulmonary disease; ACEI, angiotensin-converting enzyme inhibitor; ARB, angiotensin receptor blocker; BB, beta-blocker; CCB, calcium channel blocker.

**Table 2:** Incidence rates of metformin-associated lactic acidosis (MALA) in patients with chronic kidney disease (CKD) by CKD stages.

	Before Propensity Score Matching (PSM)					After PSM		
	No. of Metformin-Treated Patients	MALA Cases (N [%])	IR ^a^ (95% CI)	IRR (95% CI)	*p*-Value ^b,c^	No. of Metformin-Treated Patients	MALA Cases (N [%])	IR ^a^ (95% CI)
All	6818	27 (0.4)	1.22 (0.80–1.77)	N/A	N/A	536	2 (0.4)	1.35 (0.16–4.90)
**CKD stages**								
Mild CKD ^d^	5463	10(0.2)	0.56 (0.26–1.03)	REF	REF	328	1 (0.3)	1.10 (0.02–6.15)
CKD 3a	704	5 (0.7)	2.18 (0.71–5.08)	3.90 (1.33–11.42)	0.021	97	1 (1.0)	3.47 (0.08–19.3)
CKD 3b	401	3 (0.7)	2.42 (0.49–7.07)	4.34 (1.19–15.76)	0.054	72	0 (0.0)	N/E
CKD 4	146	4 (2.7)	10.44 (2.85–26.7)	18.72 (5.87–59.71)	<0.001	39	0 (0.0)	N/E
CKD 5	104	5 (4.8)	20.24 (6.57–47.2)	86.21 (29.47–252.21)	<0.001	N/E	N/E	N/E

^a^ Per 1000 person-years. ^b^ *p*-value for the incidence rate ratio with mild CKD as the reference group. ^c^ *p*-value calculated using Fisher’s exact two-sided test. ^d^ Mild CKD is defined as CKD stages 1 to 2. Abbreviations: IR, incidence rate; CI, confidence interval; IRR, incidence rate ratio; N/A, not applicable; REF, reference; N/E, not estimated due to too few patients left after PSM.

## Data Availability

Data is contained within the article and Supplementary Materials.

## Supplementary Materials

The following supporting information can be downloaded at: https://www.mdpi.com/article/10.3390/ph17101369/s1, Table S1. Yearly trends of prescribing antidiabetic drugs in patients with chronic kidney disease (CKD) over the study period by CKD stages.
