# Supplementary material for: Trends in Antidiabetic Drug Use and Safety of Metformin in Diabetic Patients with Varying Degrees of Chronic Kidney Disease from 2010 to 2021 in Korea: Retrospective Cohort Study Using the Common Data Model
Source: Pharmaceuticals (Basel). 2024 Oct 14;17(10):1369. doi: 10.3390/ph17101369 (PMC11510110; doi:10.3390/ph17101369)
Supplement: Supplementary file 1 [file pharmaceuticals-17-01369-s001.zip › pharmaceuticals-3219320-supplementary.pdf]

# Supplementary Material

# Trends of Antidiabetic Drug Use and Safety of Metformin in Diabetic Patients with Varying Degrees of Chronic Kidney Disease from 2010 to 2021 in Korea: Retrospective Cohort Study by Using the Common Data Model

**Sung Hwan Joo†, Seungwon Yang†, Suhyun Lee†, Seok Jun Park, Taemin Park, Sang Youl Rhee, Jae Myung Cha, Sandy Jeong Rhie, Hyeon Seok Hwang*, Yang Gyun Kim*, Eun Kyoung Chung***

**Table S1.** Yearly trends of prescribing antidiabetic drugs in patients with chronic kidney disease (CKD) over the study period by CKD stages: A, mild CKD (i.e., stages 1 to 2); B, CKD stage 3a; C, CKD stages 3b to 5.[a]

A. Mild CKD (i.e., stages 1 to 2)

| | 2010 | 2011 | 2012 | 2013 | 2014 | 2015 | 2016 | 2017 | 2018 | 2019 | 2020 | 2021 | P-value[b] |
|---|---|---|---|---|---|---|---|---|---|---|---|---|---|
| **MET** | 1,436 (40.6) | 335 (34.5) | 332 (32.4) | 290 (28.4) | 319 (29.8) | 398 (29.0) | 408 (29.4) | 473 (31.0) | 523 (32.8) | 656 (33.0) | 442 (34.4) | 91 (30.8) | 0.493 |
| **SUs** | 919 (26.0) | 195 (20.1) | 220 (21.5) | 172 (16.9) | 193 (18.0) | 209 (15.2) | 222 (16.0) | 237 (15.6) | 225 (14.1) | 294 (14.8) | 179 (14.0) | 49 (16.6) | 0.01 |
| **Insulin** | 377 (10.7) | 156 (16.0) | 125 (12.2) | 145 (14.2) | 113 (10.5) | 132 (9.6) | 130 (9.4) | 152 (10.0) | 190 (11.9) | 207 (10.4) | 136 (10.6) | 48 (16.3) | 0.868 |
| **DPP4is** | 304 (8.6) | 210 (21.6) | 258 (25.2) | 295 (28.9) | 360 (33.6) | 405 (29.5) | 379 (27.4) | 415 (27.2) | 426 (26.7) | 483 (24.4) | 317 (24.7) | 71 (24.1) | 0.122 |
| **TZDs** | 331 (9.4) | 13 (1.3) | 52 (5.1) | 104 (10.2) | 79 (7.4) | 77 (5.6) | 59 (4.3) | 50 (3.3) | 55 (3.4) | 64 (3.2) | 37 (2.9) | 4 (1.4) | 0.008 |
| **SGLT2is** | 0 (0.0) | 0 (0.0) | 0 (0.0) | 0 (0.0) | 0 (0.0) | 147 (10.7) | 178 (12.8) | 192 (12.6) | 176 (11.0) | 276 (13.9) | 170 (13.2) | 32 (10.8) | < 0.001 |
| **Glinide** | 117 (3.2) | 40 (4.0) | 30 (2.8) | 15 (1.4) | 7 (0.6) | 16 (1.2) | 19 (1.4) | 14 (0.9) | 7 (0.4) | 4 (0.2) | 5 (0.4) | 0 (0.0) | 0.270 |
| **AGIs** | 168 (4.8) | 63 (6.5) | 38 (3.7) | 14 (1.4) | 8 (0.7) | 3 (0.2) | 9 (0.6) | 4 (0.3) | 0 (0.0) | 3 (0.2) | 2 (0.2) | 0 (0.0) | < 0.001 |

B. CKD stage 3a

| | 2010 | 2011 | 2012 | 2013 | 2014 | 2015 | 2016 | 2017 | 2018 | 2019 | 2020 | 2021 | P-value[b] |
|---|---|---|---|---|---|---|---|---|---|---|---|---|---|
| **MET** | 276 (37.6) | 47 (26.6) | 44 (25.6) | 48 (27.8) | 48 (28.7) | 35 (27.3) | 40 (26.5) | 44 (24.4) | 48 (32.6) | 55 (27.8) | 42 (33.9) | 9 (34.6) | 0.644 |
| **SUs** | 219 (29.9) | 33 (18.6) | 35 (20.4) | 34 (19.7) | 26 (15.6) | 14 (10.9) | 26 (17.2) | 27 (15.0) | 20 (13.6) | 25 (12.6) | 20 (16.1) | 2 (7.7) | 0.009 |
| **Insulin** | 91 (12.4) | 44 (24.9) | 33 (19.2) | 23 (13.3) | 25 (15.0) | 21 (16.4) | 22 (14.6) | 20 (11.1) | 22 (15.0) | 22 (11.1) | 9 (7.3) | 5 (19.2) | 0.072 |
| **DPP4is** | 50 (6.8) | 39 (22.0) | 39 (22.7) | 44 (25.4) | 45 (27.0) | 44 (34.4) | 49 (32.4) | 67 (37.2) | 38 (25.8) | 66 (33.3) | 36 (29.0) | 6 (23.1) | < 0.001 |
| **TZDs** | 53 (7.2) | 4 (2.3) | 13 (7.6) | 20 (11.6) | 18 (10.8) | 9 (7.0) | 8 (5.3) | 7 (3.9) | 9 (6.1) | 6 (3.0) | 5 (4.0) | 0 (0.0) | 0.017 |
| **SGLT2is** | 0 (0.0) | 0 (0.0) | 0 (0.0) | 0 (0.0) | 0 (0.0) | 5 (3.9) | 6 (4.0) | 14 (7.8) | 10 (6.8) | 23 (11.6) | 9 (7.3) | 4 (15.4) | < 0.001 |
| **Glinide** | 43 (5.5) | 16 (8.3) | 8 (4.4) | 5 (2.8) | 3 (1.8) | 6 (4.5) | 5 (3.2) | 2 (1.1) | 1 (0.7) | 0 (0.0) | 0 (0.0) | 0 (0.0) | 0.002 |
| **AGIs** | 44 (6.0) | 10 (5.6) | 8 (4.6) | 4 (2.3) | 5 (3.0) | 0 (0.0) | 0 (0.0) | 1 (0.6) | 0 (0.0) | 1 (0.5) | 3 (2.4) | 0 (0.0) | 0.009 |

C. CKD stage 3b to 5

| | 2010 | 2011 | 2012 | 2013 | 2014 | 2015 | 2016 | 2017 | 2018 | 2019 | 2020 | 2021 | P-value[b] |
|---|---|---|---|---|---|---|---|---|---|---|---|---|---|
| **MET** | 332 (30.1) | 51 (20.8) | 53 (20.0) | 45 (19.6) | 47 (20.4) | 39 (18.5) | 41 (22.2) | 25 (16.9) | 23 (16.9) | 29 (17.6) | 11 (20.4) | 0 (0.0) | < 0.001 |
| **SUs** | 293 (26.6) | 48 (19.6) | 61 (23.0) | 35 (15.2) | 43 (18.6) | 35 (16.6) | 32 (17.3) | 30 (20.3) | 22 (16.2) | 22 (13.3) | 9 (16.7) | 5 (20.0) | 0.59 |
| **Insulin** | 285 (25.8) | 79 (32.2) | 73 (27.8) | 58 (25.2) | 52 (22.5) | 44 (20.8) | 38 (20.5) | 29 (19.6) | 30 (22.1) | 34 (20.6) | 8 (14.8) | 7 (29.0) | 0.30 |
| **DPP4is** | 56 (5.1) | 44 (18.0) | 46 (17.4) | 56 (24.4) | 66 (28.6) | 68 (32.2) | 49 (26.5) | 47 (31.8) | 47 (34.6) | 52 (31.5) | 19 (35.2) | 9 (36.0) | < 0.001 |
| **TZDs** | 60 (5.4) | 6 (2.4) | 19 (7.2) | 28 (12.2) | 17 (7.4) | 16 (7.6) | 15 (8.1) | 7 (4.7) | 8 (5.9) | 12 (7.3) | 3 (5.6) | 0 (0.0) | 0.11 |
| **SGLT2is** | 0 (0.0) | 0 (0.0) | 0 (0.0) | 0 (0.0) | 0 (0.0) | 5 (2.4) | 7 (3.8) | 10 (6.8) | 4 (2.9) | 14 (8.5) | 4 (7.4) | 4 (16.0) | < 0.001 |
| **Glinide** | 110 (9.1) | 33 (11.9) | 24 (8.3) | 17 (6.9) | 10 (4.2) | 11 (5.0) | 7 (3.7) | 5 (3.3) | 4 (2.9) | 5 (2.9) | 1 (1.8) | 2 (7.4) | 0.06 |
| **AGIs** | 77 (7.0) | 17 (6.9) | 13 (4.9) | 8 (3.5) | 6 (2.6) | 4 (1.9) | 3 (1.6) | 0 (0.0) | 2 (1.5) | 2 (1.2) | 0 (0.0) | 0 (0.0) | 0.01 |

[a] Data are presented as count (%).
[b] P-value from the Cochrane-Armitage test to assess the presence of time trend
Abbreviations: MET, metformin; SUs, sulfonylureas; DPP4is, dipeptidyl peptidase-4 inhibitors; TZDs, thiazolidinediones; SGLT2is, sodium-glucose cotransporter-2 inhibitors; Glinide, meglitinide; AGIs, alpha-glucosidase inhibitors.
